# Diene Valepotriates from *Valeriana glechomifolia* Prevent Lipopolysaccharide-Induced Sickness and Depressive-Like Behavior in Mice

**DOI:** 10.1155/2015/145914

**Published:** 2015-06-11

**Authors:** Liz G. Müller, Milene Borsoi, Eveline D. Stolz, Vivian Herzfeldt, Alice F. Viana, Ana Paula Ravazzolo, Stela Maris K. Rates

**Affiliations:** ^1^Programa de Pós Graduação em Ciências Farmacêuticas, Universidade Federal do Rio Grande do Sul, 90610-000 Porto Alegre, RS, Brazil; ^2^Programa de Pós Graduação em Neurociências, Universidade Federal do Rio Grande do Sul, 90046-900 Porto Alegre, RS, Brazil; ^3^Faculdade de Veterinária, Universidade Federal do Rio Grande do Sul, 91540-000 Porto Alegre, RS, Brazil

## Abstract

*Valeriana glechomifolia*, a native species from southern Brazil, presents antidepressant-like activity and diene valepotriates (VAL) contribute to the pharmacological properties of the genus. It is known that depression can develop on an inflammation background in vulnerable patients and antidepressants present anti-inflammatory properties. We investigated the effects of VAL (10 mg/kg, p.o.) on sickness and depressive-like behaviors as well as proinflammatory cytokines (IL-1*β* and TNF-*α*) and BDNF expression in the cortex of mice exposed to a 5 min swimming session (as a stressful stimulus) 30 min before the *E. coli* LPS injection (600 *µ*g/kg, i.p.). The forced swim + LPS induced sickness and depressive-like behaviors, increased the cortical expression of IL-1*β* and TNF-*α*, and decreased BDNF expression. VAL was orally administered to mice 1 h before (pretreatment) or 5 h after (posttreatment) *E. coli* LPS injection. The pretreatment with VAL restored the behavioral alterations and the expression of cortical proinflammatory cytokines in LPS-injected animals but had no effects on BDNF expression, while the posttreatment rescued only behavioral alterations. Our results demonstrate for the first time the positive effects of VAL in an experimental model of depression associated with inflammation, providing new data on the range of action of these molecules.

## 1. Introduction

Major depressive disorder (MDD) is a recurrent and incapacitating mood disorder being related to high mortality and morbidity [1]. Despite the fact that the accepted “monoamine hypothesis” contributed to the comprehension about the neurobiology of mood disorders, the pathogenesis of MDD has not been completely elucidated yet [2]. Thus, the identification of novel biological targets and pathways that may play a role in MDD pathophysiology is required.

In this regard, several studies have been pointing to the association of the immune system activation with MDD [3, 4]. Of note, depressed patients display elevated plasma levels of proinflammatory cytokines such as interleukin- (IL-) 1*β*, IL-6, and tumor necrosis factor-alpha (TNF-*α*) [2, 5–7] as well as increased expression of proinflammatory cytokines in frontal cortex [8]. Also, some studies showed that the association of anti-inflammatories to conventional antidepressants increased the efficacy of these drugs [9–11].

Preclinical studies have been demonstrating that systemic administration of* Escherichia coli* lipopolysaccharide (LPS) to rodents results in behavioral time-dependent changes related to increased peripheral and central proinflammatory cytokines production [3, 12, 13]. It is known that LPS-injected rodents present behavioral signs of sickness (such as decreased locomotor and exploratory activities) that are followed by depressive-like behavior [3]. The switch from sickness to depression occurs after the activation of indoleamine 2,3-dioxygenase, which culminates in decreased central serotonin levels [14].

Recently, evidences have suggested the involvement of disrupted neuroplasticity in MDD pathophysiology [3, 15]. Furthermore, hippocampal neurogenesis and neurotrophins (especially the brain derived neurotrophic factor, BDNF) expression are thought to be necessary for the behavioral effects of antidepressants [16, 17]. In addition, some authors demonstrated that intraperitoneal administration of LPS to rodents is correlated with decreased BDNF levels in the hippocampus and cortex [13, 18, 19].

Noteworthy, the administration of antidepressants [18, 20] to animals subjected to the above mentioned model of depression prevents the development of sickness and depressive-like behavior. Interestingly, the antidepressant potential of anti-inflammatory drugs has been demonstrated in the forced swimming test in models of depression related to stress in rats [21]. Also, the anti-inflammatory properties of natural products in LPS-injected rodents have been shown by several studies [22–24].

In line with these observations, some studies have been pointing to the anti-inflammatory properties of the* Valeriana* genus, which is widely known by its sedative and anxiolytic properties [25], representing a new approach to the therapeutic use of the genus. The anti-inflammatory potential of species such as* V. wallichii* [26],* V. amurensis* [27], and* V. officinalis*, which prevents the sickness and depressive-like behavior in rats submitted to a model of depression related to inflammation [28], has been demonstrated. Furthermore, some authors have already shown the antidepressant potential of those species [25, 29, 30].

In this sense, our research group has been investigating the pharmacological properties of* V. glechomifolia* Meyer, one species native to southern Brazil that is especially enriched in valepotriates [31]. Valepotriates are nonglycosylated carbocyclic iridoids, comprising a family of terpenes [32] that contribute to the pharmacological properties of the genus [33–36]. The antidepressant potential of diene valepotriates was demonstrated by our research group as well as its action on noradrenergic and dopaminergic neurotransmission [37], suggesting a dual-action mechanism distinct of most of the conventional antidepressants.

Considering the above mentioned data, in the present study we investigated the effects of a diene valepotriates fraction (VAL) obtained from* V. glechomifolia* on sickness and depression-like behavior triggered by intraperitoneal administration of* E. coli* LPS, in mice previously submitted to a forced swimming session as a stressful stimulus [13]. We also investigated VAL effects on the cortical expression of proinflammatory cytokines (IL-1*β* and TNF-*α*) and BDNF. This experimental protocol was chosen on the basis of the concept that internal and external stressors interact, resulting in an illness state that causes an allostatic overload [38, 39].

## 2. Materials and Methods

### 2.1. Drugs and Chemicals

For the extract characterization, grade HPLC acetonitrile and methanol were purchased from Merck (Germany). For the behavioral experiments, the following drugs were used: imipramine from Henrifarma (Porto Alegre, Brazil) and lipopolysaccharide (LPS) from* Escherichia coli* serotype 0111:B4 from Sigma Chemical Co. (St. Louis, MO, USA). All chemicals were obtained in the highest grade.

### 2.2. Plant Material

*Valeriana glechomifolia* above-ground and below-ground material was collected during its flowering stage in the region of São José dos Ausentes, state of Rio Grande do Sul, Brazil. The collection was authorized by SISBIO-IBAMA (protocol n° 29495-1). The identification was performed by Dr. M. Sobral (Universidade Federal de São João del-Rei, state of Minas Gerais, Brazil) and a voucher specimen (Sobral, 7733) was deposited in the Herbarium of the Universidade Federal do Rio Grande do Sul (ICN), Brazil.

### 2.3. VAL Extraction and Characterization by HPLC

To obtain the VAL fraction, 100 g (dry weight) of dried and powdered plant material was submitted to supercritical CO_2_ (SCCO_2_) extraction, using a Pilot Equipment as described elsewhere [37, 40, 41]. The conditions of the extraction were 40°C, 90 bar, SCCO_2_ flow rate through the extraction vessel: 6.67 × 10^−4^ kg s^−1^. The SCCO_2_ extraction recovery was 2.96 g%.

The VAL fraction was dissolved in HPLC grade methanol and filtered (0.22 *μ*m pore size, Merck) before the analysis by HPLC according to a method previously described [37, 41, 42], using Shimadzu HPLC system and Waters Nova-Pack C18 column (4 mm, 3.9 × 150 mm i.d. with Waters Nova-Pack C-18 guard column, 60 Å, 3.9 × 20 mm). The isocratic mobile phase consisted of acetonitrile and water (50 : 50 v/v); flow rate of 1 mL/min; UV detection at 254 nm. All diene valepotriates were quantified in terms of mg of valtrate equivalent/g extract. The VAL fraction was suspended in saline with 1% of polysorbate 80 (vehicle) prior to use.

### 2.4. Animals

Male CF1 mice (25–30 g) were from Fundação Estadual de Produção e Pesquisa em Saúde, Rio Grande do Sul, Brazil. Animals were housed in plastic cages (17 × 28 × 13 cm) at 23° ± 1°C under a 12-hour light/dark cycle, with food and water provided* ad libitum*. Experiments were approved by Animal Care Local Ethical Committee (CEUA-UFRGS; protocol n° 22648) and were conducted in accordance with Brazilian law [43–45] and European Communities Council Directive of 24 November 1986 (86/609/EEC).

### 2.5. Experimental Design

The experimental protocol was carried out according to Viana and coworkers [13] with minor modifications. The animals (*n* = 8–11 mice/group) were submitted to a prestressful stimulus (6 min forced swimming session, water at 23 ± 1°C) 30 min before receiving LPS from* E. coli* (600 *μ*g/kg, i.p.) or vehicle (0.9% NaCl solution, 10 mL/kg, i.p.). The animals were submitted to behavioral tests 6 h and 24 h after the LPS injection.

**Figure 1:**
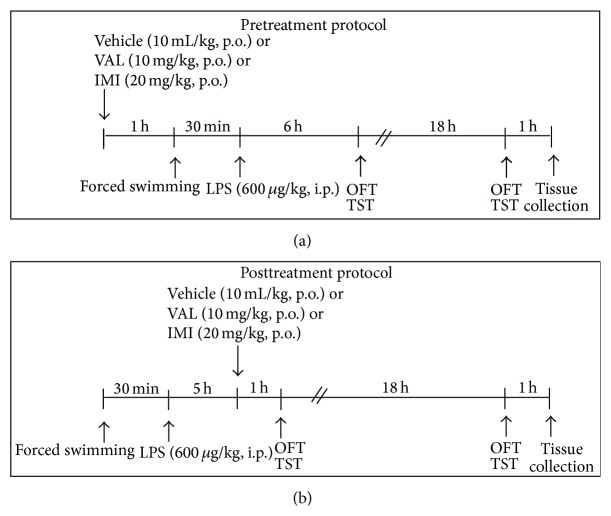
Experimental timeline and design. OFT: open field test; TST: tail suspension test.

In order to verify the effects of VAL on forced swim + LPS-induced behavioral and neurochemical alterations, independent groups of mice were treated with VAL (10 mg/kg, p.o.) or vehicle (0.9% NaCl solution with 1% of polysorbate 80) 1 h before the forced swimming session (pretreatment protocol, in order to evaluate their preventive potential) or 5 h after LPS injection (posttreatment protocol, in order to evaluate their therapeutic potential). The antidepressant imipramine (IMI) was used as a positive control (20 mg/kg, p.o.). The doses of VAL and IMI were determined on the basis of previous studies from our group [37]. Mice were sacrificed by cervical dislocation 1 h after the final behavioral test for quantitative reverse transcription polymerase chain reaction (RT-qPCR) analysis. A naïve group was included for RT-qPCR analysis. A schematic overview of the experimental design is shown in Figure 1.

### 2.6. Behavioral Paradigms

#### 2.6.1. Open Field Test (OFT)

To assess the effects of the forced swimming session + LPS treatment (as well as the effects of VAL and IMI before and after treatment) on the locomotor activity, mice were evaluated in the OFT, 6 or 24 h after LPS administration. Animals were individually placed in an acrylic box (40 × 30 × 30 cm), with the floor being divided into 24 equal squares. The number of squares crossed with the four paws (crossing) was recorded in a 6 min session.

#### 2.6.2. Tail Suspension Test (TST)

Mice depression-like behavior following the forced swimming session + LPS treatment (as well as the effects of VAL and IMI before and after treatment) was evaluated by using the TST as previously described by Steru and coworkers [46] with minor modifications. Immediately after being submitted to the OFT (6 or 24 h after LPS injection), the animals were suspended by the tail 60 cm above the floor using adhesive tape (1 cm from the tip of the end). The time during which mice remained immobile was recorded (in seconds) during the last 4 min of a total 6 min session [47].

### 2.7. Quantitative Reverse Transcription Polymerase Chain Reaction (RT-qPCR) for Quantification of IL-1*β*, TNF-*α*, and BDNF mRNA Expression

Mice cortices were collected and immediately immersed in liquid nitrogen and stored at −80°C until use. Total RNA was extracted using TRIzol Reagent (Invitrogen), according to the manufacturer's instructions. The concentration and purity of the RNA were assessed at 260 nm and 260/280 nm absorbance measurements, respectively. Also, its integrity was evaluated by using agarose gel electrophoresis stained with ethidium bromide. Complementary DNA (cDNA) was synthesized from 2 *μ*g of total RNA using a High Capacity cDNA Transcription kit (Applied Biosystems Inc., Foster City, CA) in a 10 *μ*L reaction. After obtaining the cDNA, samples were stored at −20°C. IL-1*β*, TNF-*α*, and BDNF expression was carried out through fluorescence-based real-time PCR, by amplifying 100 ng of cDNA (in duplicates) using TaqMan-based chemistry with specific primers, FAM-labeled probes (Assays-by-Demand, Life Technologies) for mouse IL-1*β* (#Mm00434228_m1), TNF-*α* (#Mm00443260_g1), and BDNF (#Mm00432069_m1) and VIC-labeled glyceraldehyde-3-phosphate dehydrogenase (GAPDH) (Assays-by-Demand, Life Technologies, #Mm99999915_g1) as the endogenous control for normalization. Amplifications were carried out in a thermal-cycler (StepOne Plus, Applied Biosystems) for 70 cycles; the fluorescence was collected at each amplification cycle and the data were analyzed using the 2^−ΔΔCt^ method for relative quantification. Expression of the target genes was calibrated against conditions found in naïve mice.

### 2.8. Statistical Analysis

Data from behavioral experiments and RT-qPCR were analyzed by two- or one-way ANOVA, respectively, followed by Student-Newman-Keuls test when appropriate. The statistical procedures were performed using the Sigma Stat software 2.03 (Jandel Scientific Corporation). The level for statistical significance was set as* p* < 0.05. The results are given as mean ± S.E.M.

## 3. Results

### 3.1. VAL Fraction Characterization

**Figure 2:**
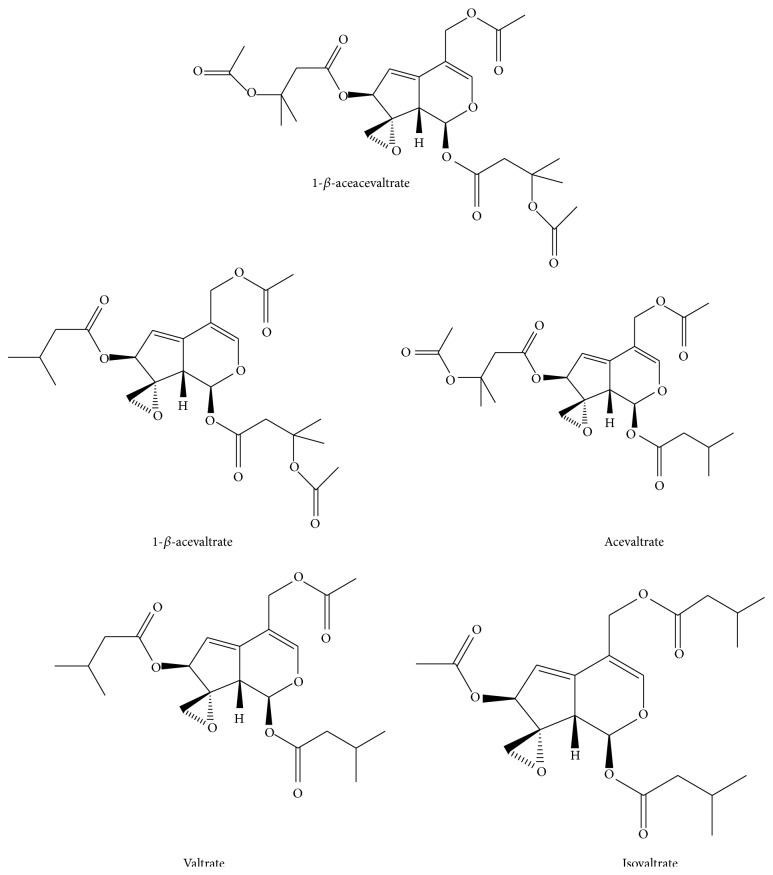
Diene valepotriates found in* Valeriana glechomifolia*.

The characterization of the VAL fraction by HPLC revealed that valtrate was the diene valepotriate present in larger quantity (643 ± 56 mg/g), followed by acevaltrate (172 ± 34 mg/g), 1-*β*-acevaltrate (87 ± 9 mg/g), 1-*β*-aceacevaltrate (39 ± 5 mg/g), and isovaltrate (37 ± 6 mg/g). Results are expressed in mean ± S.D. HPLC chromatograms of the VAL fraction have already been published [41]. The chemical structures of each diene valepotriate are presented in Figure 2.

### 3.2. Effects of VAL and IMI on Forced Swim + LPS-Induced Sickness and Depression-Like Behavior

**Figure 3:**
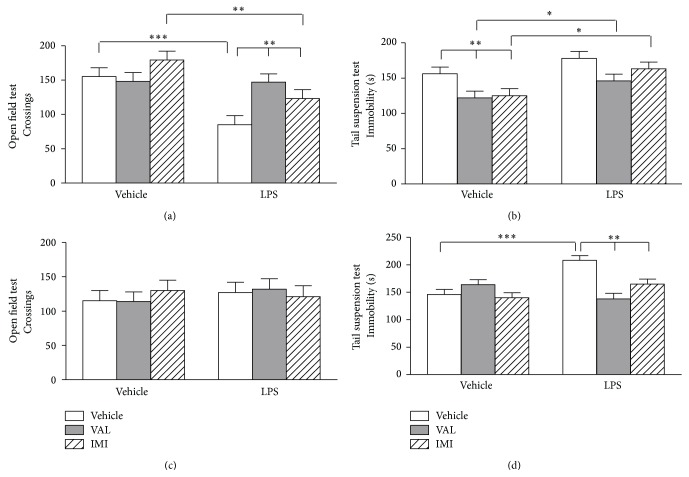
Effects of diene valepotriates from* V. glechomifolia* (VAL) pretreatment on sickness and depressive-like behavior induced by a stressful stimulus (6 min forced swimming session) +* E. coli* LPS injection in mice. The animals were orally treated with VAL (10 mg/kg, p.o.) or imipramine (IMI, used as a positive control, 20 mg/kg, p.o.) 1 hour before being exposed to the forced swim + LPS and were evaluated in the open field and tail suspension tests 6 h (Panels (a) and (b)) and 24 h after (Panels (c) and (d)) the immune challenge. Each column represents the mean ± S.E.M (*n* = 8–12 mice/group). Two-way ANOVA,* post hoc* Student-Newman-Keuls test. ^*∗*^ *p* < 0.05; ^*∗∗*^ *p* < 0.01; ^*∗∗∗*^ *p* < 0.001.

The effects of VAL or IMI pretreatment protocol on forced swim + LPS-induced behavioral alterations are presented in Figure 3. The forced swim + LPS administration elicited a significant (*p* < 0.001) decrease in mice locomotor activity at 6 h after the injection (Figure 3(a)), which was prevented by mice pretreatment with VAL and IMI [*F*_pretreatment_(1,59) = 17.589,* p* < 0.001; *F*_forced  swim+LPS_(1,59) = 4.037,* p* < 0.05; *F*_pretreatment×forced  swim+LPS_(1,59) = 4.307,* p* < 0.05]. The oral administration of VAL and IMI significantly (*p* < 0.01) decreased mice immobility time in the TST 6 h after vehicle injection when compared to the vehicle-vehicle treated group and this effect was not observed when the animals were pretreated with VAL or IMI and received the LPS injection (Figure 3(b)) [*F*_pretreatment_(1,59) = 11.556,* p* < 0.001; *F*_forced  swim+LPS_(1,59) = 5.922,* p* < 0.01; *F*_pretreatment×forced  swim+LPS_(1,59) = 0.398,* p* = 0.674]. The locomotor activity of the animals returned to basal levels at 24 h (Figure 3(c)) [*F*_pretreatment_(1,59) = 0.0001;* p* = 0.992; *F*_forced  swim+LPS_(1,59) = 0.122,* p* = 0.885; *F*_pretreatment×forced  swim+LPS_(1,59) = 1.431,* p* = 0.248]. The forced swim + LPS administration significantly (*p* < 0.001) increased mice immobility time in the TST 24 h after the injection (Figure 3(d)) when compared to vehicle-vehicle treated animals, while VAL and IMI pretreatment prevented the forced swim + LPS-induced immobility injection [*F*_pretreatment_(1,59) = 6.601;* p* < 0.05; *F*_forced  swim+LPS_(1,59) = 4.721,* p* < 0.05; *F*_pretreatment×forced  swim+LPS_(1,59) = 10.863,* p* < 0.001].

**Figure 4:**
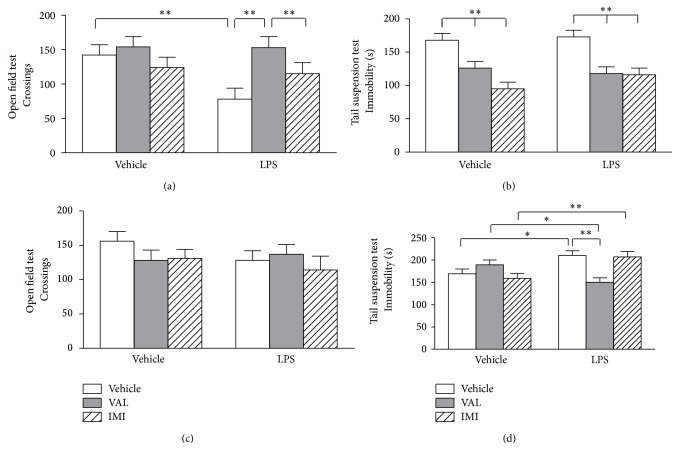
Effects of diene valepotriates from* V. glechomifolia* (VAL) posttreatment on sickness and depressive-like behavior induced by a stressful stimulus (6 min forced swimming session) +* E. coli* LPS injection in mice. The animals were orally treated with VAL (10 mg/kg, p.o.) or imipramine (IMI, used as a positive control, 20 mg/kg, p.o.) 5 hours after being exposed to the forced swim + LPS and were evaluated in the open field and tail suspension tests 6 h (Panels (a) and (b)) and 24 h after (Panels (c) and (d)) the immune challenge. Each column represents the mean ± S.E.M (*n* = 8–12 mice/group). Two-way ANOVA,* post hoc* Student-Newman-Keuls test. ^*∗*^ *p* < 0.05; ^*∗∗*^ *p* < 0.01.

The effects of VAL or IMI posttreatment on forced swim + LPS-induced behavioral alterations are shown in Figure 4. Mice posttreatment with VAL, but not with IMI, prevented the forced swim + LPS-induced decrease in the locomotor activity assessed by the OFT 6 h after LPS injection (Figure 4(a)) [*F*_forced  swim+LPS_(1,58) = 9.755,* p* < 0.01; *F*_post  treatment_(1,58) = 8.3691,* p* < 0.001; *F*_forced  swim+LPS×post  treatment_(1,59) = 0.918,* p* = 0.406]. Mice orally posttreated with VAL and IMI presented a significant (*p* < 0.01) decrease in the immobility time in the TST 6 h after vehicle or LPS injection (Figure 4(b)) [*F*_forced  swim+LPS_(1,59) = 0.479,* p* = 0.492; *F*_post  treatment_(1,58) = 21.114,* p* < 0.001; *F*_post  treatment×forced  swim+LPS_(1,59) = 0.920,* p* = 0.406]. The locomotor activity of the animals returned to basal levels 24 h after LPS injection, as can be seen in Figure 4(c) [*F*_forced  swim+LPS_(1,59) = 0.135,* p* = 0.715; *F*_post  treatment_(1,59) = 0.896,* p* = 0.415; *F*_post  treatment×forced  swim+LPS_(1,59) = 2.036,* p* = 0.141]. Only VAL posttreatment was able to prevent (*p* < 0.01) the forced swim + LPS-induced increase in the immobility time in the TST 24 h after LPS injection [*F*_forced  swim+LPS_(1,59) = 3.405,* p* = 0.071; *F*_post  treatment_(1,59) = 1.749,* p* = 0.184; *F*_post  treatment×forced  swim+LPS_(1,59) = 9.876,* p* < 0.001].

### 3.3. Effects of VAL and IMI on Forced Swim + LPS-Induced Alterations in the Expression of IL1-*β*, TNF-*α*, and BDNF Expression in the Cortex of Mice

**Figure 5:**
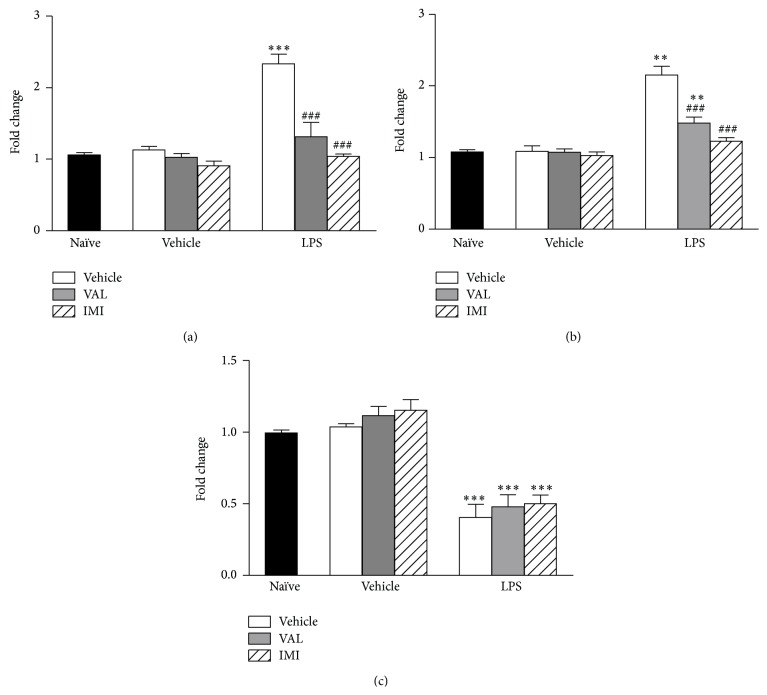
Effects of diene valepotriates from* V. glechomifolia* (VAL) pretreatment on the alterations in the cortical expression of IL1-*β* (Panel (a)), TNF-*α* (Panel (b)), and BDNF (Panel (c)) induced by a stressful stimulus (6 min forced swimming session) +* E. coli* LPS injection in mice. The animals were orally treated with VAL (10 mg/kg, p.o.) or imipramine (IMI, used as a positive control, 20 mg/kg, p.o.) 1 hour before being exposed to the forced swim + LPS and the tissues were collected 25 h later. Each column represents the mean (*n* = 4 mice/group). One-way ANOVA,* post hoc* Student-Newman-Keuls test. ^*∗∗*^ *p* < 0.01; ^*∗∗∗*^ *p* < 0.001 versus Naïve group; ### versus Vehicle+LPS-treated group.

The effects of pretreatment with VAL and IMI on forced swim + LPS-induced changes in the cortical expression of IL1-*β*, TNF-*α*, and BDNF are shown in Figure 5. The forced swim + LPS elicited a significant increase in the expression of IL1-*β* (*p* < 0.001) (Figure 5(a)) and TNF-*α* (*p* < 0.01) (Figure 5(b)) in the cortex of mice, while VAL and IMI pretreatment prevented this effect [IL1-*β*: *F*(6,28) = 23.76,* p* < 0.001; TNF-*α*:* F*(6,28) = 32.21,* p* < 0.001]. Cortical BDNF expression was significantly (*p* < 0.001) reduced by the forced swim + LPS (Figure 5(c)). Nevertheless, mice pretreatment with VAL and IMI was not effective in preventing this effect [*F*(6,28) = 25.48,* p* < 0.001].

**Figure 6:**
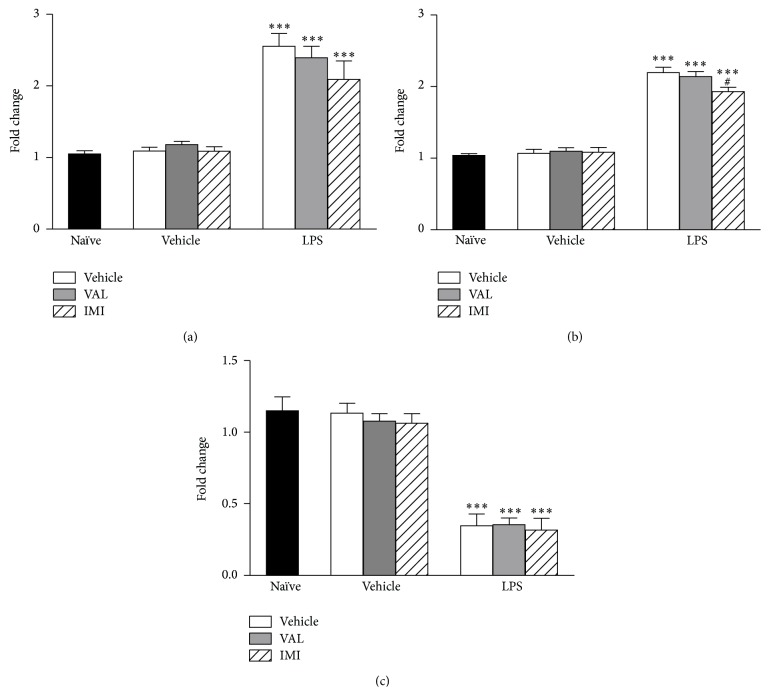
Effects of diene valepotriates from* V. glechomifolia* (VAL) posttreatment on the alterations in the cortical expression of IL1-*β* (Panel (a)), TNF-*α* (Panel (b)), and BDNF (Panel (c)) induced by a stressful stimulus (6 min forced swimming session) +* E. coli* LPS injection in mice. The animals were orally treated with VAL (10 mg/kg, p.o.) or imipramine (IMI, used as a positive control, 20 mg/kg, p.o.) 5 hours after being exposed to the forced swim + LPS and the tissues were collected 25 h later. Each column represents the mean (*n* = 4 mice/group). One-way ANOVA,* post hoc* Student-Newman-Keuls test. ^*∗∗∗*^ *p* < 0.001 versus Naïve group; ^#^ *p* < 0.05 versus Vehicle + LPS-treated group.

The effects of mice posttreatment with VAL and IMI on forced swim + LPS-induced alterations in the expression of IL1-*β*, TNF-*α*, and BDNF in mice cortex are depicted in Figure 6. The forced swim + LPS significantly increased (*p* < 0.001) the expression of IL1-*β* (Figure 6(a)) and TNF-*α* (Figure 6(b)), while a significant (*p* < 0.001) decrease in BDNF expression was found in the cortex of mice. The posttreatment with VAL or IMI did not prevent these alterations in the expression of proinflammatory cytokines [IL1-*β*:* F*(5,28) = 23.89,* p* < 0.001; TNF-*α*: *F*(5, 28) = 83.54,* p* < 0.001] or BDNF [*F*(5,58) = 31.84,* p* < 0.001].

## 4. Discussion

The present study demonstrated the positive effects of* Valeriana glechomifolia* diene valepotriates (VAL) in an animal model of depression that correlates the activation of inflammatory pathways with the manifestation of depression-like behavior. Also, the antidepressant-like effect of VAL was accompanied by normalization in the expression of cortical proinflammatory cytokines in* E. coli* LPS-injected animals previously submitted to a 6 min swimming session as a stressful stimulus. These findings are in accordance with Neamati and coworkers' study [28], who reported that a hydroalcoholic extract of* V. officinalis* prevented the development of sickness and depression-like behavior in ovalbumin sensitized rats, which is an animal model of depression associated with inflammation. In line with these findings, accumulating evidence points to the antidepressant and anti-inflammatory potential of* Valeriana* species [25–27, 29, 30, 48].

Sickness behavior is a usual response to infection characterized by endocrine, autonomic, and behavioral changes triggered by the activation of the peripheral innate immune system [3]. In rodents, the sickness behavior can be detected by a reduction in locomotor activity and exploratory behaviors [49]. Herein, we demonstrated that the administration of LPS to mice previously submitted to a 6 min swimming session significantly decreased the locomotor activity 6 h after LPS, indicating the development of sickness behavior. There were no differences between the immobility time of the vehicle-treated and LPS-treated groups in the TST 6 h after the peripheral immune challenge, corroborating with the hypothesis that depression-like behavior develops over a background of sickness and peaks 24 h later [3]. No alterations in mice locomotor activity were observed 24 h after the immune challenge, confirming that the increased immobility time in the TST 24 h after LPS is due to the manifestation of depression-like behavior.

The pre- and posttreatment with VAL resulted in normalization of behavioral alterations elicited by LPS in mice previously submitted to a forced swimming session. On the other hand, mice posttreatment with IMI was not able to restore the forced swim + LPS-triggered effects. In fact, there are several studies in the literature demonstrating the preventive, but not the therapeutic, effects of antidepressants or natural products on LPS-induced behavioral alterations [13, 23, 24, 50]. However, other authors [18] demonstrated that mice posttreatment with IMI restored the depressive-like behavior elicited by the LPS injection. This could be due to the different experimental protocols used, since the animals used in our study were submitted to a forced swimming session before LPS injection (600 *μ*g/kg) and received an oral administration of IMI, whereas Ferreira Mello and coworkers [18] performed the LPS injection only (500 *μ*g/kg) and the administration of IMI by the i.p. route.

In our experiments, the antidepressant-like effects of VAL and IMI were accompanied by a decrease in the expression of IL-1*β* and TNF-*α* in mice cortex, which is a cerebral area related to the pathophysiology of depression [51, 52]. Consistently, the pivotal proinflammatory cytokines involved in sickness and depression-related behaviors following infection are IL-1*β* and TNF-*α* [3]. Thus, the effects of VAL and IMI on these cytokines may explain the positive results of VAL and IMI pretreatment in the behavioral tests. Of note, Jacobo-Herrera and coworkers [53] demonstrated that sesquiterpenes from* V. officinalis* showed inhibitory activity against the nuclear factor NF-kB in* in vitro* studies. These findings are particularly relevant, since this nuclear factor has been considered to play a role in the proinflammatory signaling pathway, mainly in the expression of proinflammatory genes including cytokines, such as IL-1*β* and TNF-*α* [54] and is activated upon the interaction of Toll-like receptors with the LPS [55]. Considering that the valepotriates comprise a large family of terpenes [32], we may suggest that these compounds decrease the production of proinflammatory cytokines by inhibiting the NF-kB activation and modulating the activation of cytokine signaling, which results in the decrease of cortical cytokines expression and, consequently, normalization of behavioral alterations. In line with our findings, some* ex vivo* studies demonstrated that antidepressants decrease the levels of proinflammatory cytokines in the serum and brain of LPS-injected rodents [18, 20]. These data are supported by* in vitro* studies demonstrating the anti-inflammatory potential of antidepressants by using central and peripheral derived cells [56–58]. Also, the inhibition of microglia activation by terpenes has been demonstrated in an* in vitro* study [59] as well as the anti-inflammatory potential of these molecules* in vivo* [60–62]. To the best of our knowledge, there are no previous studies regarding the anti-inflammatory properties of diene valepotriates.

Interestingly, mice posttreatment with VAL, but not with IMI, prevented the forced swim + LPS-induced behavioral alterations and had no effects on IL-1*β* and TNF-*α* expression in the cortex, while VAL pretreatment decreased the cortical expression of IL-1*β* and TNF-*α*. These findings may suggest that the effects of VAL posttreatment on behavioral alterations could be due to its action on dopaminergic and noradrenergic neurotransmissions. Our research group showed that the acute antidepressant-like effect of VAL was prevented by mice pretreatment with yohimbine (*α*2 adrenoceptor antagonist), SCH 23390 (D1 dopamine receptor antagonist), and sulpiride (D2 dopamine receptor antagonist) [37]. The findings of the present study may also suggest that VAL prevents the activation of IL-1*β* and TNF-*α* signaling cascades, but it is not able to block these cascades once they were activated. Another possibility is that other brain regions, than cortex, might be involved in the anti-inflammatory effects of VAL posttreatment.

Our results demonstrate that the stressful stimulus followed by the peripheral immune challenge decreases the cortical expression of BDNF mRNA at 24 h after LPS and these findings are in agreement with several studies that used animal models of depression associated to inflammation [13, 18–20]. However, mice before and after treatment with VAL or IMI were not effective in preventing these alterations. In fact, several authors have reported that the acute administration of conventional antidepressants, including IMI, is not able to increase the expression of BDNF [63–67]. Moreover, our results suggest that VAL could exert its effects in the cortex acting mainly by inhibiting an inflammatory pathway and not by interfering with BDNF.

## 5. Conclusion

Altogether, the results so far reinforce the antidepressant-like potential of* V. glechomifolia* diene valepotriates and demonstrate the positive effects of these compounds in an animal model that associates the activation of inflammatory pathways to depression etiology. The behavioral effects of diene valepotriates administration to the animals were accompanied by the normalization of proinflammatory cytokines expression, which brings a new focus on the range of action of these molecules.

## Abbreviations

ANOVA: Analysis of variance
BDNF: Brain derived neurotrophic factor
HPLC: High performance liquid chromatography
IL: Interleukin
IMI: Imipramine
LPS: Lipopolysaccharide
MDD: Major depressive disorder
OFT: Open field test
RT-qPCR: Quantitative reverse transcription polymerase chain reaction
S.E.M: Standard error of the mean
SCCO_2_: Supercritical CO_2_
SISBIO-IBAMA: Sistema de Autorização e Informação em Biodiversidade do Instituto Brasileiro do Meio Ambiente e dos Recursos Naturais Renováveis
TNF-*α*: Tumor necrosis factor alpha
TST: Tail suspension test
VAL: Diene valepotriates fraction.
